# Changes in Bond Strength and Topography for Y-TZP Etched with Hydrofluoric Acid Depending on Concentration and Temperature Conditions

**DOI:** 10.3390/medicina56110568

**Published:** 2020-10-28

**Authors:** Hyo-Eun Kim, Myung-Jin Lim, Mi-Kyung Yu, Kwang-Won Lee

**Affiliations:** Department of Conservative Dentistry, Jeonbuk National University School of Dentistry 567 Baekje-daero, Jeonju 54896, Korea; hwa3106@naver.com (H.-E.K.); occasio00@naver.com (M.-J.L.); mkyou102@jbnu.ac.kr (M.-K.Y.)

**Keywords:** hydrofluoric acid, hot etching, shear bond strength, surface roughness, Y-TZP

## Abstract

*Background and objectives*: This study aimed to investigate the change in bond strength between resin cement and tetragonal zirconia polycrystalline stabilized with 3 to 8 mol% yttrium oxide (Y-TZP) and observe the topographical change of the Y-TZP surface when etched with hydrofluoric acid (HF) solution under different concentration and temperature conditions. *Materials and Methods*: Non-etched sintered Y-TZP specimens under two different temperature conditions (room temperature and 70–80 °C, respectively), were used as a control, while experimental groups were etched with 5%, 10%, 20%, and 40% HF solutions for 10 min. After zirconia primer and MDP-containing resin cement were applied to the Y-TZP surface, the shear bond strength (SBS) of each experimental group was measured. *Results*: Under room temperature conditions, the highest SBS value was measured in the 40% HF etching group, representing a significant deviation from the other groups (*p* < 0.05). In the 70–80 °C tests, the 40% HF etching group also had the highest SBS value, but there was no significant difference when compared to the 20% HF etching group (*p* > 0.05). From SEM and AFM observations, the HF solution increasingly dissolved the Y-TZP surface grain structure as the concentration and application temperature rose, resulting in high surface roughness and irregularities. *Conclusions*: Pretreating with either 20% HF solution at 70–80 °C or 40% HF solution at room temperature and 70–80 °C effectively acid etched the Y-TZP surface, resulting in more surface roughness and irregularities. Accounting for the concentration and temperature conditions of the HF solution, using 40% HF solution at room temperature will result in improvements in adhesion between resin cement and Y-TZP.

## 1. Introduction

Zirconia restorations have superior mechanical properties compared with other ceramic systems used in dentistry [1,2], and accordingly, have found a number of clinical applications, including as inlays/onlays, single crown/bridges, implant abutments, orthodontic brackets, and fixed-partial dentures [3,4].

Zirconia, however, comes with a drawback. The absence of a silica glass component and the homogenous, densely packed structure of zirconia crystals make etching and silanization (and therefore durable bonding) difficult [5,6]. Strong and reliable bonding with ceramic restorations relies on chemical bonding and micro-mechanical interlocking [7,8]. Chemical bonding is achieved using a functional monomer, 10-methacryloyloxydecyl dihydrogen phosphate (MDP) [9,10,11]. According to Noriyuki et al. [12], the chemical bonds between 10-MDP and zirconia are the result of ionic and hydrogen bonding. Other research has shown that the use of a primer and resin cement containing 10-MDP results in a relatively high bond strength in zirconia restorations [13]. However, other studies [5,14] have reported that improved bond strength to zirconia treated with MDP-containing primers cannot be maintained for a long time due to the acceleration of hydrolysis at the bonding site.

Several studies have been undertaken to examine the methods and effects of increased micromechanical interlocking between zirconia restoration and luting resin cement [15,16,17]. The conventional method of increasing the micromechanical retention by airborne-particle abrasion (sandblasting) with Al_2_O_3_ or SiO_2_ powder not only weakens the zirconia restoration by causing unfavorable surface modifications such as micropores (which can act as crack initiators), but also induce the zirconia crystals to undergo an unfavorable phase transformation from tetragonal to monoclinic [18,19,20]. Consequently, this method seems poorly suited for establishing reliable zirconia/resin cement micromechanical interlocking.

Alternative means of modifying the surface of zirconia restorations to increase its micromechanical interlocking with luting resin cement have been proposed in the literature, including selective infiltration etching (SIE) [21], high-temperature chemical etching [22], and hydrofluoric acid (HF) etching [23]. Of these, HF etching has received the most attention. Until recently, the HF etching of zirconia was generally thought to be impossible. However, recent studies have shown that etching is, in fact, possible with adjustments to the various application conditions, including concentration, immersion time, and temperature [23,24]. As most studies on HF etching have been conducted at room temperature, this study was designed to investigate changes in the bond strength between resin cement and tetragonal zirconia polycrystalline stabilized with 3 to 8 mol% yttrium oxide (Y-TZP) when the HF solution was raised to 70–80 °C. This study also observed the topographical changes of Y-TZP surfaces etched with the HF solution under various concentrations and temperature conditions.

The two null hypotheses of this study were that changes to the concentration and temperature of hydrofluoric acid neither (1) increased the bonding strength between zirconia and resin cement, nor (2) caused topographical changes on the Y-TZP surface. 

## 2. Materials and Methods

The materials used in this study are listed in Table 1.

Ninety sintered Y-TZP specimens (NexxZr T, Sagemax, Federal Way, WA, USA) and resin blocks were prepared for the shear bond strength (SBS) test. Y-TZP specimens in the form of standard cylinders (12 mm in diameter, 8 mm in height) were prepared by CAD/CAM (Shanghai Machine Tool Ltd., Shanghai, Chaina) milling and sintering. The resin blocks were prepared by incremental packing (Unifil Loflo, GC Inc., Tokyo, Japan) into a cylindrical Teflon mold (3 mm in diameter, 10 mm in height) and then light-curing each increment for 20 s. 

**Figure 1:**
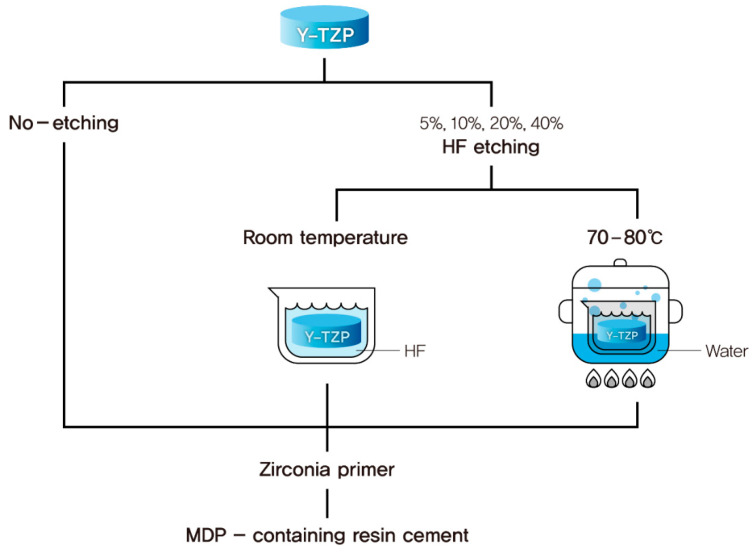
Schematic representation of the pretreatment procedures undergone by the zirconium oxide (Y-TZP) specimen following different hydrofluoric acid (HF) etching conditions.

Non-etched sintered Y-TZP specimens (*n* = 10) were used as the control. Other experimental groups were etched with varied concentrations of HF under varied temperature conditions (Figure 1). HF solutions (of 5%, 10%, 20%, and 40% concentration) were experimentally prepared using 48% HF solution (MKBH5499V, Sigma-Aldrich Co., St. Louis, MO, USA), distilled water, and an electronic scale. Under two different temperature conditions (room temperature, 70–80 °C), ten Y-TZP specimens for each experimental group were etched with 5%, 10%, 20%, and 40% HF solutions for 10 min in a plastic box. The specimens were then ‘hot etched’; a water bath was used to raise the temperature of the HF solution to 70–80 °C. After etching, the Y-TZP specimens were ultrasonically cleaned in distilled water for 10 min and then gently air-dried.

Zirconia primer (Z-prime plus, Bisco Inc., Schaumburg, IL, USA) was applied to the surface of all groups of Y-TZP for 10 s, after which all groups were air-dried for 15 s. Resin cement containing MDP (G-CEM LinkAce, GC Inc.) was then applied to all groups. Under a constant load of 5 N, light curing was performed for 3 s for tack curing, and the excess resin cement was removed using hand instruments. While maintaining the constant load, all surfaces were light-cured for 20 s. The specimens that underwent luting procedures were stored in distilled water at 36 °C for 24 h before the SBS test. 

Specimens were then mounted in the jig of a universal testing machine (Model 5543, Instron, Canton, MA, USA) and shear force (using a chisel-shaped metal rod at a constant crosshead speed of 0.5 mm/min) was applied to the specimen until failure. Load was measured by recording the force at which the failure occurred, and the SBS was calculated using the following formula: SBS (MPa) = load (N)/area (mm^2^).

One Y-TZP disk specimen from each HF etch group was rinsed with 95% ethanol, air dried, mounted on a metal stub, and coated with a gold-palladium alloy using sputter coating technology (Ion sputter E-1030, Hitachi, Tokyo, Japan). Subsequently, the structural change of each HF-etched Y-TZP surface was observed at 10,000 times magnification with a scanning electron microscope (SEM) (SU8230, Hitachi, Tokyo, Japan).

One Y-TZP disk specimen from each HF etch group was used for the morphological change and surface roughness analysis using atomic force microscopy (AFM) (Multimode-8, Bruker, Santa Barbara, CA, USA). To analyze the surface roughness of each group of HF etched Y-TZP specimens, a silicon AFM probe (k = 42 N/m, f = 320 kHz, NCHR, Nanoworld, Neuchâtel, Switzerland) was used to perform in tapping mode with a scan size of 5 × 5 µm. Five measurements were performed for each specimen and the average was determined as the mean Ra value.

Statistical analysis was performed using statistical software (SPSS Version 18.0, SPSS Inc., Chicago, IL, USA). The mean and standard deviation (SD) of SBS (*n* = 10) and surface roughness Ra values were statistically analyzed using two-way analysis of variance (ANOVA), and significant differences between the groups were determined using the pairwise multiple comparison procedure (Tukey’s test). *p*-values less than 0.05 were considered statistically significant in all tests.

## 3. Results

**Figure 2:**
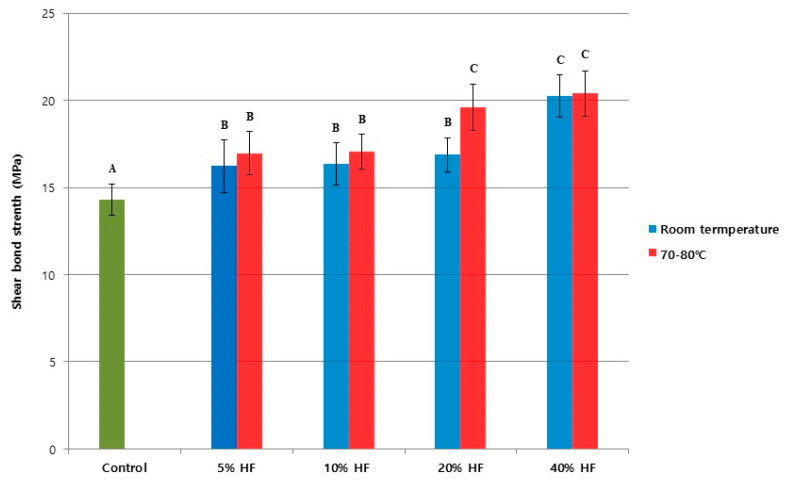
Shear bond strengths (MPa) of the control group that did not etch the surface and the experimental groups that did etch the Y-TZP surface with hydrofluoric acid under various conditions. Identical capital letters indicate that there are no statistically significant differences (*p* > 0.05).

The mean SBS values with standard deviations are presented in Figure 2. 

All HF etching groups showed significantly higher SBS values when compared to the control group (*p* < 0.05). Two-way ANOVA showed that both concentrations of the HF solution and the etching temperature had a significant effect on SBS (*p* < 0.05). Interactions between the various concentrations of HF solutions and the etching temperature conditions were also statistically significant (*p* < 0.05).

At room temperature, the highest SBS value was measured in the 40% HF etching group, representing a significant difference compared to the other groups (*p* < 0.05). No statistically significant differences were found between the 5%, 10% and 20% HF etching groups (*p* > 0.05), although these HF etching groups did show higher SBS values than the control (*p* < 0.05). Consistent with the test undertaken under room temperature conditions, at 70–80 °C, the 40% HF etching group had the highest SBS value. Here, however, there were no significant difference from the 20% HF etching group (*p* > 0.05) under these temperature conditions. Overall, the 20% HF etching group at 70–80 °C, as well as the 40% HF etching groups at both room temperature and 70–80 °C, were significantly different from the other groups (*p* < 0.05).

**Figure 3:**
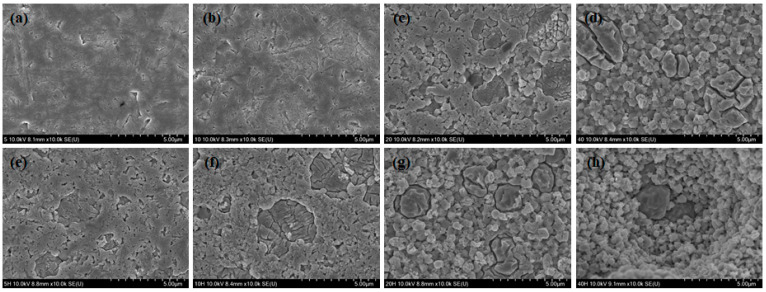
SEM images of the Y-TZP surface etched with hydrofluoric acid (HF) under various conditions: (**a**) room temperature: 5% HF; (**b**) room temperature, 10% HF; (**c**) room temperature, 20% HF; (**d**) room temperature, 40% HF; (**e**) 70–80 °C, 5% HF; (**f**) 70–80 °C, 10% HF; (**g**) 70–80 °C, 20% HF; (**h**) 70–80 °C, 40% HF.

Representative SEM images of HF etched Y-TZP specimens under different conditions are shown in Figure 3. SEM observation revealed that the 40% HF etching groups from both the room temperature and the 70–80 °C tests showed a completely different morphology compared to the control group. The dissolution of the Y-TZP surface grain structure by the HF solution increased with the concentration and application temperature, resulting in high surface roughness and irregularities. However, among the low concentration (5%, 10%) HF etching groups, the solution was not effective for etching Y-TZP surfaces regardless of temperature.

**Figure 4:**
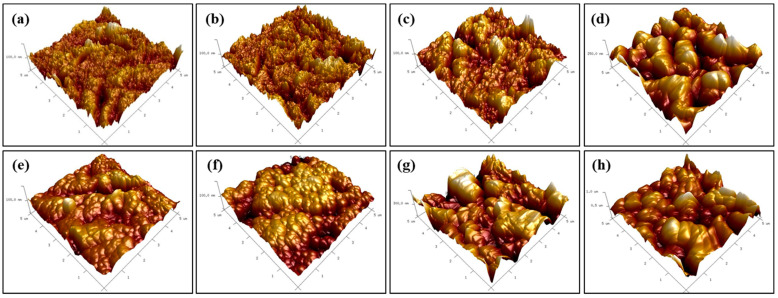
AFM images of the Y-TZP surface etched with hydrofluoric acid (HF) under various conditions: (**a**) room temperature, 5% HF; (**b**) room temperature, 10% HF; (**c**) room temperature, 20% HF; (**d**) room temperature, 40% HF; (**e**) 70–80 °C, 5% HF; (**f**) 70–80 °C, 10% HF; (**g**) 70–80 °C, 20% HF; (**h**) 70–80 °C, 40% HF.

**Figure 5:**
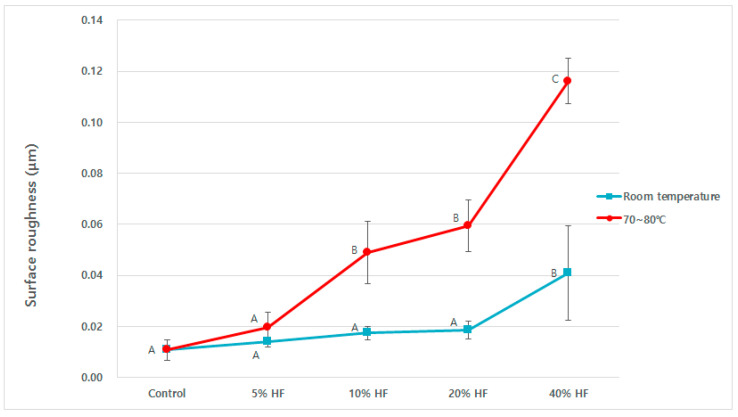
Mean surface roughness (Ra) of Y-TZP etched with hydrofluoric acid depending on the concentration and temperature conditions. Identical uppercase letters indicate no statistically significant differences (*p* > 0.05).

Representative AFM images of HF etched Y-TZP specimens under different conditions are shown in Figure 4 and Figure 5. The AFM images also revealed that the HF solution could etch the Y-TZP surface. The mean Ra values increased along with temperature in all HF etching groups. The 5% HF etching group had the least increase in the mean Ra value, the 10% and 20% HF etching groups showed similar increases, and the 40% HF etching group had the largest increase. The 40% HF etching group at room temperature showed a significantly higher Ra value, than that which was apparent in the other HF etching groups (*p* < 0.05). There was no significant difference in the mean Ra values between the 5%, 10%, and 20% HF etching groups at room temperature (*p* > 0.05). The 40% HF etching group also displayed a significantly higher mean Ra value than the other HF etching groups (*p* < 0.05) at temperatures of 70–80 °C. In these tests, unlike the room temperature tests, the 10% and 20% HF etching groups showed significantly higher mean Ra values than the 5% HF etching group (*p* < 0.05).

## 4. Discussions

Hydrofluoric acid is an inorganic compound consisting of hydrogen fluoride (HF) in water. HF is used in industrial processes such as glass etching, metal cleaning, and silicon wafer etching, and as laboratory reagents [25,26]. In dentistry, a concentration of 4–10% HF is used as a magnetic/ceramic surface conditioner [27]. HF has a distinct ability to dissolve the glass phase, leaving behind a crystalline phase of porcelain/ceramic and with a rough surface. However, it is a well-known limitation of HF that it does not affect zirconia due to its high crystallinity and lack of glass phase [5,6].

This study has shown that conventional knowledge is only partially correct. In fact, under the right conditions, HF can etch zirconia. SEM and AFM images of HF etched Y-TZP specimens revealed that an HF solution partially etched the Y-TZP superficial grain structure (in), leading to the formation of irregular grooves (increasing the inter-grain size) and nano-sized porosity (reducing the grain size). Etching can also occur inside the grains of the Y-TZP specimens, as atoms around the crystal boundary can dissolve faster than atoms inside the crystal [23,28].

There are few studies on the Y-TZP etching mechanism by HF. Flamant et al. [29] that attempt to theoretically explain the dissolution of ZrO_2_ in HF, based on the Pourbaix speciation diagrams. According to Lowalekar et al. [30], element F in a 40% HF solution dissolved zirconium oxide and yttrium oxide to form fluoride, oxide and hydroxide complexes.

In this experiment, not only at room temperature, but also how efficiently the Y-TZP surface is etched by hydrofluoric acid at 70–80 °C, was investigated. This is because it was thought that even at the same concentration, increasing the temperature would increase the surface area etched by hydrofluoric acid, resulting in an increase in micromechanical bonds. However, since hydrofluoric acid has a low boiling point, it can be easily vaporized and can be very harmful to the human body. In addition, in this experiment, since a water bath was used, the degree of etching by hydrofluoric acid was studied at a temperature of 70–80 °C lower than the boiling point of water, 100 °C.

Our study showed that a surface change and a significant increase in the mean Ra value were observed from the 10% HF etched group at 70–80 °C, but not the group etched at room temperature. It was also found that as the concentration of the HF solution increased, the higher the average Ra value rose as the temperature increased. As both the temperature and concentration increased, the HF solution became increasingly molecularly reactive, making it possible to etch the Y-TZP specimen surface much faster and more effectively. Yu et al. [31] reportedly achieved similar results by applying HF-based zirconia etching solution at high temperature. Casucci et al. [22] also reported that when etching the zirconia surface with a hot acidic solution (100 °C), the inter-particle spacing and surface roughness increased significantly. This technique can only be helpful in increasing the etching efficiency for Y-TZP up to a point; when the HF solution is heated past its boiling point, it inevitably produces extremely dangerous and potentially lethal HF vapor [32].

There is a controversy concerning whether HF etching pretreatment for Y-TZP improves the bond strength. Some studies have reported that HF etching for Y-TZP does not improve bond strength [23,24,33]. According to their research results, nano-sized porosity and irregular pattern structures were formed on the HF-etched Y-TZP surface, but the viscosity of the resin cement was not low enough, suggesting that micromechanical interlocking was not properly formed. They also suggested that HF etching induced the phase transformation from tetragonal to monoclinic structure of Y-TZP due to the low-temperature degradation associated with humid conditions [34,35]. Moreover, they concluded that the phase transformation caused by this degradation did not improve the bonding strength by reducing the physical properties of Y-TZP. Lee et al. [36], however, reported that the phase transformation from tetragonal to monoclinic structure was observed in all HF etching groups, but as the monoclinic structure of the HF etching group was smaller than that of the airborne-particle abrasion group, the HF etching itself did not induce the sufficient phase transformation to the extent that it adversely affected the bond strength to Y-TZP.

The present study showed that the HF etching pretreatment improved the bond strength to Y-TZP compared to the control. In our study, the low-viscosity resin cement penetrated into the irregularly patterned fine-sized porous structure formed after HF etching. The higher the mean Ra value, the stronger the micromechanical linkage could be formed, so the higher the bond strength was measured at a higher mean Ra value.

There was no difference in adhesive strength between the 40% HF etching groups, though the mean Ra value was higher at 70–80 °C than at room temperature. The SEM image of the 40% HF etching group at 70–80 °C showed that the Y-TZP surface was not uniformly etched, and that a part of the sample was much more deeply recessed compared to the nearby parts. Non-homogeneous etching like this would have made it difficult to achieve strong micromechanical interlocking and is the reason that the bond strength did not increase despite the growth in the mean Ra value. These results indicate that there is no advantage to dangerously increasing the temperature of hydrofluoric acid when etching Y-TZP with a 40% HF solution.

Based on these results, both the null hypotheses initially proposed were rejected. Further studies are needed to achieve a more detailed understanding of the mechanism by which HF etches the Y-TZP surface and the most suitable etching conditions (concentration, temperature, and immersion time) that will improve the bonding strength of the Y-TZP surface.

## 5. Conclusions

Within the limits of this study, pretreatment with 20% HF solution at 70–80 °C or 40% HF solution at room temperature and 70–80 °C can effectively acid-etch the Y-TZP surface, resulting in high surface roughness and irregularities. However, accounting for the concentration and temperature conditions of the HF solution, 40% HF solution at room temperature is recommended to improve the adhesion between the resin cement and Y-TZP.

## Figures and Tables

**Table 1:** Materials used, manufacturers and major components.

Material/Trade Name	Manufacturer	Main Components
Hydrofluoric acid (HF) /Hydrofluoric acid	Merck KGaA, Darmstadt, Germany	48 wt% in H_2_O
Zirconia Primer /Z-prime Plus	Bisco Inc., Schaumbrug, IL, USA	BPDM, HEMA, ethanol
MDP-containing resin cement /G-CEM LinkAce	GC Corp., Tokyo, Japan	Paste A: Fluoro-alumino-silicate glass, UDMA, dimethacrylate, Silicon dioxide Paste B: Phosphoric acid ester monomer (MDP), silicon dioxide, UDMA, dimethacrylate

Abbreviation: BPDM (biphenyl dimethacrylate), HEMA (hydroxyethyl methacrylate), UDMA (urethane dimethacrylate), MDP (10-methacryloyloxydecyl dihydrogen phosphate).
